# Pro-Inflammatory and Pro-Oxidative Changes During Nilotinib Treatment in CML Patients: Results of a Prospective Multicenter Front-Line TKIs Study (KIARO Study)

**DOI:** 10.3389/fonc.2022.835563

**Published:** 2022-02-01

**Authors:** Anna Sicuranza, Ilaria Ferrigno, Elisabetta Abruzzese, Alessandra Iurlo, Sara Galimberti, Antonella Gozzini, Luigiana Luciano, Fabio Stagno, Antonella Russo Rossi, Nicola Sgherza, Daniele Cattaneo, Corrado Zuanelli Brambilla, Cristina Marzano, Carmen Fava, Olga Mulas, Emanuele Cencini, Adele Santoni, Vincenzo Sammartano, Alessandro Gozzetti, Luca Puccetti, Monica Bocchia

**Affiliations:** ^1^ Hematology Unit, University of Siena, Azienda Ospedaliero Universitaria Senese, Siena, Italy; ^2^ S. Eugenio Hospital, Tor Vergata University, Rome, Italy; ^3^ Hematology Division, Foundation IRCCS Ca’ Granda Ospedale Maggiore Policlinico, Milan, Italy; ^4^ Department of Clinical and Experimental Medicine, University of Pisa, Pisa, Italy; ^5^ Department of Cellular Therapies and Transfusion Medicine, AOU Careggi, Florence, Italy; ^6^ Hematology Unit, AUOP Federico II, Naples, Italy; ^7^ Hematology Section and BMT Unit, Rodolico Hospital, AOU Policlinico-V. Emanuele, Catania, Italy; ^8^ Hematology and Transplantation Unit, University of Bari, Bari, Italy; ^9^ Hematology and Transplant Center, Casa Sollievo della Sofferenza Hospital, San Giovanni Rotondo, Italy; ^10^ Department of Clinical and Biological Sciences, University of Turin, Turin, Italy; ^11^ Department of Medical Sciences and Public Health, University of Cagliari, Businco Hospital, Cagliari, Italy; ^12^ Hemostasis and Thrombosis Unit, University of Siena, Azienda Ospedaliero Universitaria Senese, Siena, Italy

**Keywords:** CML, TKI, cytokines, AOEs, cardiovascular risk (CV risk)

## Abstract

Tyrosine kinase inhibitors (TKI) may offer a normal life expectancy to Chronic Myeloid Leukemia (CML) patients. However, a higher than expected incidence of arterial occlusive events (AOEs) was observed during treatment with nilotinib. We previously showed an “inflammatory status” during nilotinib that may explain the increased incidence of AOEs. Thus, we conducted this prospective KIARO study involving 186 CML patients (89 imatinib, 59 nilotinib, 38 dasatinib). Interleukin 6 (IL6), interleukin 10 (IL10), Tumor Necrosis Factor-α (TNFα), oxLDL, and high-sensitivity C-reactive protein (hs-CRP) plasma levels were measured at diagnosis and during treatment, with the aim to investigate changes in the inflammatory status favoring AOEs of each patient. Clinical and biochemical pro-atherothrombotic profiles and the 10-year SCORE chart were also evaluated. We showed a pro-inflammatory/pro-oxidative milieu increasing along treatment with nilotinib compared with imatinib or dasatinib, as demonstrated by higher hs-CRP and oxLDL levels and increased IL6/IL10 and TNFα/IL10 ratios only in nilotinib cohort. After median follow-up of 23.3 months starting from TKI, 10/186 patients (5.4%) suffered an AOE. Approximately 5/10 (50%) AOEs occurred during nilotinib treatment despite a lower 10-year SCORE and a lower median age in this subgroup. A longer follow-up is needed to further confirm the active role of nilotinib in AOEs pathogenesis.

## Introduction

The great success of tyrosine kinase inhibitor (TKI) treatment in chronic myeloid leukemia (CML) marked a decisive change in the history of the disease, offering patients a life expectancy comparable to that of the general population (1). Indeed, TKIs ensure the maintenance of a stable, long-term deep response with the possibility, in the current clinical practice, of TKI discontinuation and the ultimate goal of treatment free remission (TFR) (2, 3). On the other hand, the long-term drug toxicity, safety, and quality of life have become crucial clinical issues. Early and more recent reports underlined the increased rate of vascular adverse effects, often arterial occlusive, in newly diagnosed CML patients treated with TKIs (4–6). Among the TKIs approved for use in first line, nilotinib was the first to be associated with peripheral artery occlusive disease (PAOD) (5, 6). More recently, nilotinib, unlike imatinib, was confirmed to be followed by a significant rate of cardiovascular (CV) adverse effects, namely, ischemic ones, which were found also to increase with long-lasting treatment exposure (7, 8). As such, over the last years many studies attempted to unravel the association between arterial occlusive events (AOEs) and nilotinib. However, despite the increasing understanding of the incidence and predisposing risk factors, the pathogenesis behind these untoward serious events is still largely unknown (9). A study analyzed *in vitro* the effect of nilotinib on endothelial cells and found that the drug upregulates pro-atherogenic adhesion molecules, such as ICAM-1, VCAM-1 and E-selectin, and determines an anti-angiogenic effect which reduces proliferation, migration, and tube-formation of endothelial cells. An increased trend toward atherosclerosis development in an apoE^−/−^ mouse model treated with nilotinib was also reported (10). Another study showed that nilotinib and third generation TKI ponatinib, but not imatinib, reduce viability of human vascular endothelial cells (11). On the clinical side, the use of the Systematic Coronary Risk Evaluation (SCORE) chart (a tool for cardiovascular risk stratification proposed by the European Society of Cardiology) was suggested to estimate the risk of developing AOEs in patients treated with nilotinib (12, 13). Afterwards, several studies retrospectively investigated associations with CV profile (assessed by the SCORE tool), the occurrence of AOEs in patients with CML treated with 2nd or 3rd generation TKIs, and the role of primary prophylaxis in preventing AOEs (14, 15). More recently, low plasma levels of low-density lipoprotein cholesterol (LDL-cholesterol) were associated with a significant lower risk of AOEs in CML patients treated with nilotinib (16).

In a previous cross-sectional retrospective study, namely, 110 chronic phase (CP)-CML patients treated with either imatinib or nilotinib, our group hypothesized that an altered inflammatory status in nilotinib treated patients, together with genetic pro-atherothrombotic predisposition, might have a role in the increased incidence of AOEs (17). In the above study we demonstrated a strong association of the G (unfavorable) allele of *OLR1* gene polymorphism (encoding LOX1 receptor) and dyslipidemia with AOEs in patients treated with nilotinib. Furthermore, biochemical analyses revealed a pro-inflammatory/oxidative status in the nilotinib cohort, characterized by lower interleukin 10 (IL10) levels and higher oxidized low-density lipoprotein (oxLDL) levels compared with the imatinib cohort.

The latter data provided the rationale to prospectively study clinical and biochemical atherothrombotic profile in CML patients at diagnosis and during treatment with any of the TKIs approved in first line (imatinib, nilotinib and dasatinib), with the aim to broaden our knowledge on potential off-target effects possibly associated with AOEs.

## Patients and Methods

Consecutive newly-diagnosed CP-CML patients referred to 11 Italian hematology centers were prospectively enrolled in this observational study at the beginning of first-line treatment with either imatinib, nilotinib or dasatinib (front-line bosutinib was not yet approved at that time). TKI choice was up to the local physician decision. The KIARO (Prospective study of tyrosine Kinase Inhibitors induced pro-AtherothROmbotic status in chronic myeloid leukemia patients) study protocol was approved by the medical ethics committee of Siena University Hospital and also the medical ethics committee at each participating center. Written informed consent was obtained from all patients in accordance with the Declaration of Helsinki.

The aims of the study were: 1) to analyze inflammation status during TKI treatment by monitoring pro/anti-inflammatory cytokines and inflammatory/oxidative biochemical parameters; 2) to record AOEs; 3) to calculate the SCORE and evaluate its predictive role for AOEs; 4) to analyze possible associations of AOEs with altered inflammation status.

At enrollment, patients were stratified according to Sokal score and studied for the presence of additional cytogenetic abnormalities. At the same time the presence of CV traditional risk factors [age, sex, arterial hypertension, diabetes, total, high density lipoprotein (HDL) and LDL cholesterol levels, personal or familial history of CV events, body mass index (BMI), smoking habit] or other comorbidities was also evaluated. The ankle-brachial index (ABI) value was measured according to the ACC/AHA guidelines (18). In addition, CV clinical and biochemical parameters were monitored at specific time-points of TKI treatment (+1, +3, +6, and +12 months) and AOEs were recorded whenever they occurred. AOEs included: PAOD, acute coronary syndromes (ACS), transient ischemic attacks (TIA) or a definite ischemic stroke of atherothrombotic origin.

For each enrolled patient, serial plasma samples were collected at diagnosis and after 1, 3, 6 and 12 months of treatment, centrifuged and immediately stored at −80°C locally at each hematology unit. The frozen samples were then centralized to the hematology laboratory in Siena.

The inflammatory/oxidative status of patients was assessed measuring plasma levels of Interleukin 6 (IL6), IL10, Tumor Necrosis Factor-α (TNFα), oxLDL and high-sensitivity C-reactive protein (hs-CRP). Cytokines levels were determined by high sensitivity Quantikine enzyme-linked immunosorbent assays (ELISA) (R&D systems), according to each manufacturer’s specifications. OxLDL and hs-CRP levels were measured by ELISA (oxLDL ELISA, Immunodiagnostik AG) and an hs-CRP immunonephelometry commercial kit (Siemens, CardioPhase, Marburg, Germany), respectively. Each ELISA test was analyzed on a Mindray MR-96A ELISA reader.

### Statistical Analysis

Multivariate analysis and the Cox proportional-hazards modeling were used to evaluate the putative relations among incidence of AOEs and both measurable and dichotomic variables in the whole cohort of subsequent CP-CML patients. For this purpose, the calculated sample size to detect a significant difference in parametric and not parametric variables evaluated, with 90% power at p = 0.01 was 134 patients, if at least 14 events would occur. Accordingly, the statistical model, consisting of a formal test for interaction, was employed to determine the putative relation for all variables, namely, the type of treatment. Final validation of data was assessed by a resampling technique (exact tests in SPSS 2003 module) and discrimination analysis by the Hosmer–Lemeshow method assuming a p <0.05 as indicating a statistical significance. To estimate putative differences in the levels of each single biochemical variable we employed the Mann–Whitney U-test and the Wilcoxon test for comparisons between and within groups. The Kendall rank correlation coefficient was used to measure the relationship among measurable variables. Furthermore Shapiro–Wilk test of normality and Levene’s test for homogeneity of variances were performed to detect which variables could be examined by three-way ANOVA. All calculations were performed using the SPSS library version 27 (SPSS Inc. Chicago, IL).

## Results

### Characteristics and Treatment of Patients

Between September 2013 and September 2018, a total of 186 CP-CML patients at diagnosis were enrolled in this study. One hundred and seven patients (58%) were males and 79 (42%) were females. Overall, 89 (48%) patients started treatment with imatinib, 59 (32%) with nilotinib, and 38 (20%) with dasatinib. As could have been expected, the prevalence of 1st line treatment choice was imatinib with respect to nilotinib or dasatinib. Median age at diagnosis in the whole cohort was 60 years (range 24–90 years) and it was significantly lower in nilotinib (51 ± 1.8) and dasatinib (56 ± 3.2) cohorts compared with imatinib (69 ± 1.3; nilotinib vs imatinib p <0.001, dasatinib vs imatinib p <0.001). Sokal score was low in 62 patients (33%), intermediate in 85 patients (46%), and high in 39 patients (21%). Additional cytogenetic abnormalities were reported for 18/186 patients (9.7%). The median follow-up since CML diagnosis was 23.3 months (range 12–64.6 months). All the characteristics of the patients are reported in **Table 1**.

**Table 1:** Patients’ characteristics.

PATIENTS’ CHARACTERISTICS	Whole cohort (n = 186)	IMATINIB (n = 89)	NILOTINIB (n = 59)	DASATINIB (n = 38)
**Median age at diagnosis (range)**	60 years (24–90 yr)	69 years (32–90 yr)	51 years (24–88 yr)	56 years (32–79 yr)
**Sex**				
** Male**	107 (58%)	54 (60.7%)	33 (56%)	20 (52.6%)
** Female**	79 (42%)	35 (39.3%)	26 (44%)	18 (47.4%)
**Sokal score**				
** High**	39/186 (21%)	21/89 (23.6%)	10/59 (17%)	8/38 (21%)
** Intermediate**	85/186 (46%)	45/89 (50.6%)	24/59 (40.7%)	16/38 (42%)
** Low**	62/186 (33%)	23/89 (25.8%)	25/59 (42.3%)	14/38 (37%)
**Additional cytogenetic abnormalities**	18/186 (9.7%)	8/89 (9%)	4/59 (6.8%)	6/38 (15.8%)
**Median follow-up since diagnosis**	23.3 months (12–64.6 mos)	21 months (12–62.7 mos)	24 months (12–64.5 mos)	27 months (12–59.8 mos)

### Cardiovascular Risk and SCORE at Study Entry

CV risk factors and history of CV disease at enrollment in the whole cohort and according to different TKI are detailed in **Table 2**. The mean 10-year SCORE (S) for the whole cohort (S = 8.3 ± 4.6) was in line with that estimated in a general age- and sex-matched reference population (S = 8.1 ± 5.2; p = 0.094). Furthermore, the prevalence of CV risk factors and/or history of major adverse cardiovascular events (MACE) in our whole cohort of CML patients resulted comparable to that of the general Italian population (combined and cumulative data 13.4% vs 13.9%; p = 0.113) (19). The 10-year SCORE in patients assigned to nilotinib was significantly lower compared with that of patients assigned to imatinib or dasatinib (nilotinib S = 6.8 ± 2.8, imatinib S = 9.5 ± 4.8, dasatinib S = 8.2 ± 3.3; nilotinib vs imatinib p = 0.02, nilotinib vs dasatinib p = 0.043). This distribution can be explained by the choice, in the current clinical practice, of other second generation TKIs rather than nilotinib for patients with known CV risk factors at diagnosis, including age.

**Table 2:** Traditional CV risk factors at enrollment.

Traditional CV risk factors	Whole cohort (n = 186)	IMATINIB (n = 89)	NILOTINIB (n = 59)	DASATINIB (n = 38)
** none**	50/186 (27%)	19/89 (21.3%)	23/59 (39%)	8/38 (21%)
** 1**	55/186 (30%)	22/89 (24.7%)	22/59 (37.3%)	11/38 (29%)
** 2**	47/186 (25%)	27/89 (30.4%)	8/59 (13.5%)	12/38 (31.6%)
** >2**	34/186 (18%)	21/89 (23.6%)	6/59 (10.2%)	7/38 (18.4%)
**Diabetes mellitus**	28/186 (15%)	16/89 (18%)	1/59 (1.7%)	11/38 (29%)
**Smoking**	30/186 (16%)	15/89 (17%)	8/59 (15.5%)	7/38 (18%)
**BMI ≥30**	19/186 (10%)	11/89 (12%)	4/59 (7%)	4/38 (10.5%)
**Hypertension**	86/186 (46%)	51/89 (57%)	19/59 (32%)	16/38 (42%)
**Hypercholesterolemia**	51/186 (27%)	31/89 (35%)	12/59 (20%)	8/38 (21%)
**Familiarity**	54/186 (29%)	27/89 (30%)	14/59 (28%)	13/38 (34%)
**CV events previously than CML diagnosis**	20/186 (11%)	17/89 (19%)	0/59 (0%)	3/38 (8%)
**10-year SCORE (S)**	S = 8.3 ± 4.6	S = 9.5 ± 4.8	S = 6.8 ± 2.8	S = 8.2 ± 3.3

### Biochemical Parameters, Blood Pressure and ABI Monitoring

Data regarding CV clinical and biochemical parameters are reported in **Supplemental Tables S1**, **S2**. No statistically significant differences were appreciated for total cholesterol, HDL-cholesterol and triglycerides levels in the three cohorts at any time point. On the other hand, we observed significantly increased LDL-cholesterol levels only in the nilotinib cohort at 3 (F = 4.960, p = 0.009) and 12 months (F = 4.726, p = 0.011) of treatment compared with imatinib and dasatinib cohorts, regardless of the concomitant use of CV medications (F = 2.160, p = 0.085). Arterial blood pressure and glycemia values did not undergo significant changes during treatment both in the whole cohort and among the three sub-cohorts (**Table S2**). ABI values were collected only in 74/186 enrolled patients; however, no significant changes in ABI were found along the treatment and across the three TKIs (F = 2.010, p = 0.094).

### Pro-/Anti-Inflammatory Cytokines Monitoring

Median cytokine (IL6, IL10, and TNFα) levels in the whole cohort and in the three TKI subgroups of patients are reported in **Supplemental Tables S3A**–**C**.

**Figure 1:**
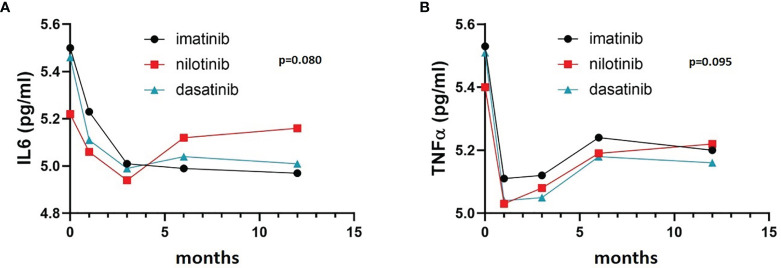
IL6 and TNFα trends in the three cohorts. Three-way ANOVA. IL6 **(A)** and TNFα **(B)** levels were high at diagnosis and decreased during the first 12 months of treatment, without any statistically significant difference between the three treatment arms (F= 2.190, p= 0.095 and F= 2.380 p= 0.080 respectively).

TNFα and IL6 levels were high at diagnosis, presumably reflecting the inflamed status caused by the disease, and then they both decreased during the first 12 months of treatment, apparently without any statistically significant difference between the three treatment arms (**Figures 1A, B**) (F = 2.190, p = 0.095 and F = 2.380, p = 0.080 respectively).

**Figure 2:**
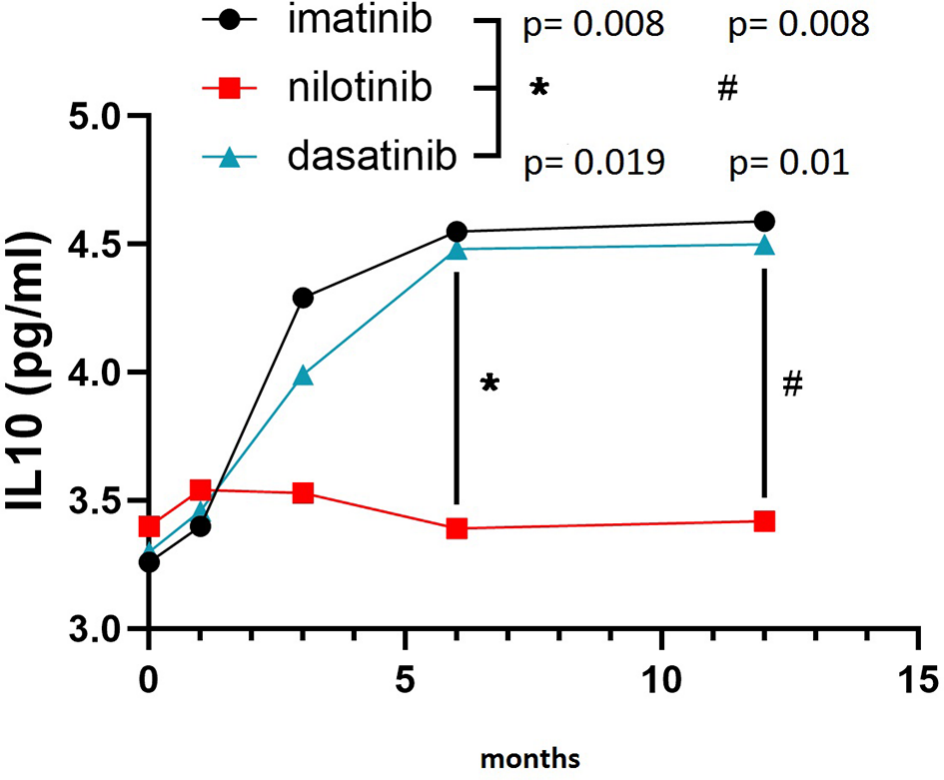
IL10 levels in the three cohorts. Three-way ANOVA. IL10 levels were higher after 6 and 12 months of imatinib (F=5.530, p=0.008 and F=5.620, p=0.008, respectively) and dasatinib (F=4.160, p=0.019 and F=4.910, p=0.01, respectively) in comparison with nilotinib. Symbols "*" and “#” are referring to statistical differences reported with p values of IL10 levels at 6 (*) and 12 (#) months of treatment in imatinib and dasatinib groups compared to nilotinib.

**Figure 3:**
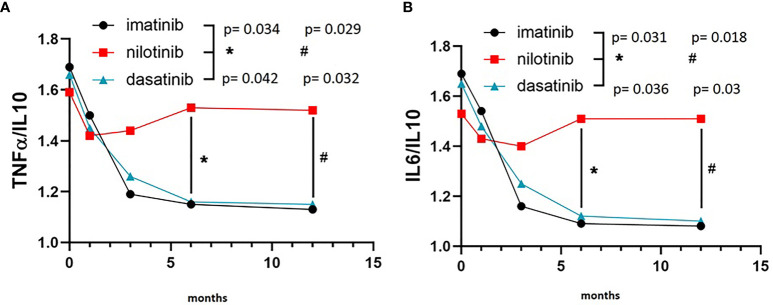
TNFα/IL10 and IL6/IL10 ratios in the three cohorts. **(A)** Three-way ANOVA. TNFα/IL10 ratio was significantly higher in nilotinib cohort at 6 and 12 months compared with imatinib (F=3.870, p=0.034 at 6 months and F=4.010, p=0.029 at 12 months) and dasatinib (F=3.620, p=0.042 at 6 months and F=3.960, p=0.032 at 12 months). **(B)** Three-way ANOVA. IL6/IL10 ratio was significantly higher in nilotinib cohort compared with imatinib (F=3.990, p=0.031 at 6 months and F=4.210, p=0.018 at 12 months) and dasatinib (F=3.800, p=0.036 at 6 months and F=4.020, p=0.03 at 12 months). Symbols "*" and “#” are referring to statistical differences reported with p values of TNFα/IL10 ratio **(A)** at 6 (*) and 12 (#) months of treatment in nilotinib group compared to imatinib and dasatinib. In **(B)** symbols "*" and “#” are referring to statistical differences reported with p values of IL6/IL10 ratio at 6 (*) and 12 (#) months of treatment in nilotinib group compared to imatinib and dasatinib.

On the other hand, IL10 levels were comparable among the three TKI cohorts at baseline, but showed a remarkably different evolution during treatment. In fact, IL10 levels were significantly higher after 6 and 12 months of imatinib (F = 5.530, p = 0.008 and F = 5.620, p = 0.008, respectively) and dasatinib (F = 4.160, p = 0.019 and F = 4.910, p = 0.01, respectively) in comparison with nilotinib (**Figure 2**). To explore the balance between pro- (TNFα and IL6) and anti-inflammatory (IL10) cytokines, we calculated the ratios TNFα/IL10 and IL6/IL10. TNFα/IL10 ratio was significantly higher in nilotinib cohort at 6 and 12 months compared with imatinib (F = 3.870, p = 0.034 at 6 months and F = 4.010, p = 0.029 at 12 months) and dasatinib (**Figure 3A**) (F = 3.620, p = 0.042 at 6 months and F = 3.960, p = 0.032 at 12 months). As well, IL6/IL10 ratio was significantly higher in nilotinib cohort compared with imatinib (F = 3.990, p = 0.031 at 6 months and F = 4.210, p = 0.018 at 12 months) and dasatinib (F = 3.800, p = 0.036 at 6 months and F = 4.020, p = 0.03 at 12 months) (**Figure 3B**). Overall, these results suggest a TKI-driven pro-inflammatory status in nilotinib treated patients. In addition, partial available results regarding cytokine levels at 18 and 24 months of treatment, confirm the persistence of this pro-inflammatory cytokines imbalance (data not shown).

### OxLDL and hs-CRP Monitoring

**Figure 4:**
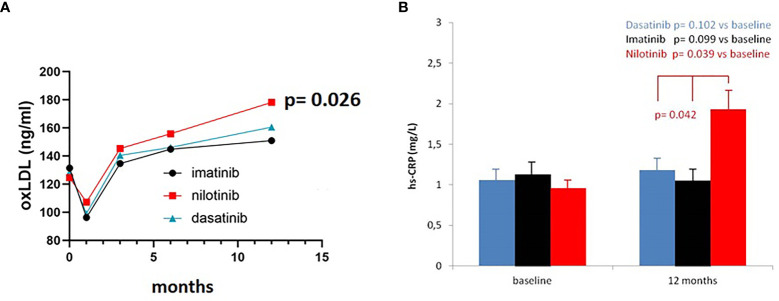
OxLDL and Hs-CRP levels in the three cohorts. **(A)** Three-way ANOVA. OxLDL levels were comparable between the three treatment arms for the first 6 months. At 12 months oxLDL levels increased in the nilotinib cohort (F= 4.030, p=0.026), compared to imatinib and dasatinib subgroups. **(B)** Three-way ANOVA. Hs-CRP levels were comparable between the three treatment arms at baseline. At 12 months hs-CRP levels increased in the nilotinib cohort (F= 3.590, p=0.042), compared to imatinib and dasatinib subgroups.

OxLDL levels were comparable between the three treatment arms for the first 6 months. At the 12 months’ time point we detected a significant increase of oxLDL levels in the nilotinib cohort (F = 4.030, p = 0.026), which we did not observe in imatinib and dasatinib subgroups (**Figure 4A**). This increase of oxLDL in nilotinib treated patients was not correlated with the level of LDL at baseline or during treatment (whole cohort: baseline r = 0.16, p = 0.094, 12 months r = 0.19, p = 0.084; nilotinib treated baseline r = 0.18, p = 0.090, 12 months r = 0.13, p = 0.112; imatinib treated: baseline r = 0.19, p = 0.085, 12 months r = 0.22, p = 0.071; dasatinib treated: baseline r = 0.14, p = 0.101, 12 months r = 0.20, p = 0.081).

We did not find significant differences in hs-CRP levels across the study during imatinib or dasatinib treatment (F = 2.920, p = 0.091 and F = 3.110, p = 0.083) whereas statistically significant higher levels were observed in nilotinib cohort at 12 months (F = 3.590, p = 0.042) (**Figure 4B**).

### Arterial Occlusive Events (AOEs)

After a median follow up of 23.3 months from the beginning of TKI treatment, 10 patients (5.4%) suffered an AOE, namely: 6 ACS, 2 PAOD, 1 TIA, and 1 stroke. Characteristics of these patients are summarized in **Table 3**.

**Table 3:** Patients who experienced AOEs.

Patient	Age	SOKAL score	10-year SCORE (S)	Previous AOE	Event	TKI at the moment of the AOE	Treatment time to event (months)
**#1**	64	intermediate	7	no	ACS	nilotinib	15.2
**#2**	49	low	5	no	ACS	nilotinib	21.1
**#3**	52	low	5	no	ACS	nilotinib	51
**#4**	44	intermediate	6	no	ACS	nilotinib	24.9
**#5**	74	intermediate	5	yes	ACS	nilotinib**	24.7
**#6**	66	intermediate	9	yes	TIA	imatinib	6
**#7**	77	intermediate	11	no	PAOD	imatinib	10.6
**#8**	68	high	6	yes	stroke	dasatinib*	17.1
**#9**	73	intermediate	6	yes	PAOD	bosutinib*	23.1
**#10**	82	intermediate	7	no	ACS	bosutinib*	16.1

*second-line; **third-line.

ACS, acute coronary syndromes; TIA, transient ischemic attacks; PAOD, peripheral artery occlusive disease.

Five events (50%) occurred in patients treated with nilotinib, either in first line (4 patients) or in third line (1 patient, after failure following brief treatment with imatinib and dasatinib). Notably, the median age of these 5 patients was 52 years (range 44–74 years). The median duration of exposure to nilotinib before the occurrence of the events was 24.7 months (range 15.2–51 months).

Of the five remaining patients who suffered an AOE, two were receiving first-line treatment with imatinib, while three were on second-line treatment after switching from imatinib to bosutinib (n = 2) or dasatinib (n = 1). Three out of these five patients had experienced an AOE before the diagnosis of CML.

In this subgroup of 10 patients experiencing an AOE, we observed a trend of increased IL6 and TNFα median values both at diagnosis and at each time point evaluated, compared with the remaining no-AOE patients. Instead, IL10 and oxLDL had similar median concentrations in both AOE and no-AOE cohorts, except for oxLDL at 12 months which resulted higher in patients who experienced AOEs (F = 4.450, p = 0.016). However, due to the low number of observed events, a formal statistical analysis for any association between AOEs and pro/anti-inflammatory cytokines levels was not possible.

### Multivariate and Relation Analyses

In the multivariate analysis we found a significant association between nilotinib treatment and changes in IL10 levels (HR 1.29, 95%CI 1.04–1.96 p <0.05), IL6/IL10 (HR 1.28, 95%CI 1.08–1.93 p <0.05) and TNFα/IL10 ratios (HR 1.21, 95%CI 1.03–1.90 p <0.05), oxLDL (HR 1.39, 95%CI 1.18–2.04 p <0.01) and LDL-cholesterol levels (HR 1.22, 95%CI 1.08–1.99 p <0.05).

Furthermore, a significant inverse relation between IL10 and oxLDL levels was found in nilotinib treated subjects starting from 3 months of treatment (r = −0.44, p <0.01). Regarding AOEs, nilotinib treatment showed a 3.1 times increased risk (HR 3.1, 95%CI 2.6–4.4 p <0.001). This result was confirmed also by three-way ANOVA only for nilotinib in comparison to the other TKIs (F = 5.030, p = 0.009). The 10-year SCORE was not predictive in the whole cohort (HR 0.6, 95%CI 0.33–0.94 p = 0.094) or in any subgroup (imatinib HR 0.8, 95%CI 0.49–1.03 p = 0.067; nilotinib HR 0.4, 95%CI 0.29–0.76 p = 0.112, dasatinib HR 0.6, 95%CI 0.37–0.92 p = 0.082).

## Discussion

The KIARO study represents the first prospective real life study in CP-CML patients demonstrating the occurrence of a pro-inflammatory and pro-oxidative status during nilotinib treatment when compared with imatinib and dasatinib. The latter evidence could play a crucial role in the pathogenesis of the AOEs observed more frequently in CML patients receiving nilotinib.

Nowadays, TKIs represent the standard cure for CML patients, allowing them a life expectancy in line with the general population. In this context, the occurrence of AOEs, particularly during nilotinib treatment, urged clinicians to review first-line therapeutic choices and to investigate the presence of traditional CV risk factors at CML diagnosis in order to minimize TKI-associated CV toxicity. However, very few data on a possible active role of any TKI, and particularly nilotinib, in the pathogenesis of AOEs were produced, without prospective studies exploring this issue. We first hypothesized, in a retrospective cross-sectional study on 110 CML patients, that an increased pro-inflammatory and oxidative status documented only in nilotinib patients may have a pathogenic role in the higher incidence of vascular events. In the KIARO study we confirmed and reinforced the data from our retrospective analysis. In particular, we prospectively showed a pro-inflammatory milieu which significantly increased over time in nilotinib treated patients compared with imatinib or dasatinib, as demonstrated by the higher levels of hs-CRP and the increased IL6/IL10 and TNFα/IL10 ratios observed only during nilotinib. Although hs-CRP has been demonstrated to reflect an inflammatory state related mostly to IL6 increase (20), our findings suggest that in nilotinib treated patients the inflammatory state is due to the imbalance between pro and anti-inflammatory cytokines. Indeed, the anti-inflammatory cytokine IL10 levels remain unchanged during nilotinib while significantly increase during imatinib and dasatinib (**Figure 2**). Notably, IL6/IL10 imbalance has been previously associated with incidence and severity of CV events in patients with acute coronary syndrome while it has been demonstrated that the overexpression of IL10 exerts an inhibitory effect on the atherosclerotic plaque formation (21).

We also observed a progressive increase in oxLDL levels during nilotinib treatment, but not during imatinib or dasatinib. The combination of inflammatory and oxidative mechanisms that are closely related in atherogenesis and atherothrombotic complications (22, 23), may eventually be responsible for nilotinib-associated endothelial activation and the consequent expression of pro-atherogenic adhesion molecules (10) with effects on macrophages and increased LOX-1 expression by these same cells (24). LOX-1, then, mediates oxLDL accumulation in the intima with atherosclerotic plaque growth but it also increases transcription and activity of proteases, responsible for plaque instability and rupture (25–28) In addition to the pathogenetic aspect, the relevance of the link between inflammatory mechanisms and lipid oxidation is also demonstrated by the reduction of clinical events as evidenced by the outcome of recent intervention trials where a specific anti-inflammatory therapy has been associated with statin treatment (29).

During the median observation time of 23 months, 10 patients (5.4%) experienced an AOE and 5/10 (50%) were on nilotinib. Despite the clear experimental evidence of a pro-inflammatory and pro-oxidative imbalance in the nilotinib subgroup, and the prevalence of AOEs in the nilotinib cohort, due to the overall low number of events a formal statistical analysis significance for any association between AOEs and type of TKI or pro and anti-inflammatory cytokine levels was not possible. A plausible explanation for lower-than-expected AOEs resides in an accurate first-line TKI selection by physicians. Indeed, the most recent ELN guidelines recommend considering CV risk factors and previous CV history at the time of TKI choice (30). As a consequence, patients at higher CV risk were rarely treated with nilotinib, as reflected by their lower baseline 10-year SCORE index compared with the imatinib and dasatinib cohorts. The strategic selection of TKI treatment according to the age of patients may also have mitigated the effect of nilotinib-induced pro-inflammatory status and consequent risk of AOEs. Indeed, we found a significantly lower median age in nilotinib and dasatinib cohorts compared with imatinib.

It is worth noting that in the nilotinib cohort, 5/59 patients (8.5%) experienced an AOE, which occurred after a median TKI exposure of 24.7 months. This is actually comparable to that reported in the literature in non-selected nilotinib treated patients, suggesting that the pro-inflammatory and pro-oxidative status could contribute over time independently from the baseline CV risk. Based on these observations, we hypothesize that, in this cohort of nilotinib patients with a lower 10-year SCORE, a longer observational time could reveal the occurrence of other events and that the evaluation of inflammatory markers including pro/anti-inflammatory cytokines should be performed for an extended treatment time. Our hypothesis of a correlation between pro-inflammatory nilotinib exposure and risk of AOEs finds a confirmation in the 10-year update of the ENESTnd trial, where after a longer follow up a significantly higher incidence of all kinds of cardiovascular adverse events, including AOEs, were documented in both 300 mg bid and 400 mg bid nilotinib cohorts compared with imatinib. Moreover, even after a Framingham CV-risk score stratification, nilotinib-treated patients in the low-risk category showed higher rates of CV events after the first 5 years of treatment compared with imatinib (8, 31).

The results of the KIARO study, prospectively comparing the AOEs outcome with the 3 TKIs first registered for front-line treatment, reinforce the idea that the choice of first-line TKI therapy should take into account both disease characteristics and personalized baseline CV profile of the patients. This could drive clinicians toward the most adequate and safe therapy considering as ultimate goals both the possibility of reaching a successful TFR and minimizing the occurrence of AOEs. Given that, when the drug of choice is nilotinib, the selection of patients based on the CV SCORE may be not sufficient to reduce the incidence of AOEs, since in our study we did not find differences in this parameter between nilotinib-treated patients who did or did not experienced an AOE. The key of this discrepancy could reside in the occurrence of a nilotinib-induced pro-atherothrombotic and pro-inflammatory status over time which may override the baseline low CV risk. Thus, regardless of pre-treatment CV risk, a careful and constant CV profile monitoring remains mandatory when nilotinib is employed as first-line treatment. Indeed, it could be important to monitor LDL levels to keep their value below 70 mg/dl, as suggested by recent data reporting an increased risk of AOEs in nilotinib-treated patients with LDL levels >70 mg/dl (16).

A longer clinical follow-up of the patients in the KIARO study, together with the prolonged monitoring of the pro-oxidative and pro-inflammatory behavior during treatment and possibly during TFR, may provide the confirmative evidence on the active role of nilotinib in the pathogenesis of adverse events of atherothrombotic origin.

## Data Availability Statement

The original contributions presented in the study are included in the article/**Supplementary Material**. Further inquiries can be directed to the corresponding author.

## Ethics Statement

The studies involving human participants were reviewed and approved by the Siena medical ethics committee. The patients/participants provided their written informed consent to participate in this study.

## Author Contributions

ASi and MB designed the study and wrote the manuscript. EA, AI, SG, AnG, LL, FS, ARR, NS, DC, CF, and OM enrolled the patients and collected clinical data and biological samples. CZB, EC, ASa, VS, and AIG collected clinical data and prepared the database of patients. ASi, IF, and CM performed the laboratory tests. LP performed the statistical analysis. All authors listed have made a substantial, direct, and intellectual contribution to the work and approved it for publication.

## Funding

The study was partially funded by a grant from the AIRC (IG 15826) and the Sienail onlus (Associazione Italiana Leucemie, Linfomi e Mieloma, sezione di Siena).

## Conflict of Interest

The authors declare that the research was conducted in the absence of any commercial or financial relationships that could be construed as a potential conflict of interest.

## Publisher’s Note

All claims expressed in this article are solely those of the authors and do not necessarily represent those of their affiliated organizations, or those of the publisher, the editors and the reviewers. Any product that may be evaluated in this article, or claim that may be made by its manufacturer, is not guaranteed or endorsed by the publisher.
