# Supplementary material for: Pro-Inflammatory and Pro-Oxidative Changes During Nilotinib Treatment in CML Patients: Results of a Prospective Multicenter Front-Line TKIs Study (KIARO Study)
Source: Front Oncol. 2022 Feb 1;12:835563. doi: 10.3389/fonc.2022.835563 (PMC8844441; doi:10.3389/fonc.2022.835563)
Supplement: Supplementary file 1 [file Table_1.docx]

| **Total**  **cholesterol**  **[mg/dl]** | **WHOLE**  **COHORT** | **IMA**  **(n=89)** | **NILO**  **(n=59)** | **DASA**  **(n=38)** | **HDL**  **[mg/dl]** | **WHOLE COHORT** | **IMA**  **(n=89)** | **NILO**  **(n=59)** | **DASA**  **(n=38)** |
| --- | --- | --- | --- | --- | --- | --- | --- | --- | --- |
| **baseline** | 172  (78-287) | 174  (83-249) | 175  (100-233) | 147  (92-287) | **baseline** | 41  (17-105) | 40  (25-95) | 44  (17-105) | 37  (18-76) |
| **+1**  **month** | 171  (79-271) | 151  (79-224) | 193  (96-271) | 183  (116-238) | **+1**  **month** | 54  (28-93) | 52  (32-85) | 57  (33-90) | 53  (28-93) |
| **+3**  **months** | 181  (84-283) | 167  (84-259) | 210  (122-283) | 185  (117-265) | **+3**  **months** | 56  (23-124) | 55  (23-89) | 59  (37-106) | 51  (32-124) |
| **+6**  **months** | 180  (80-312) | 159  (80-245) | 212  (134-312) | 182  (140-273) | **+6**  **months** | 54  (19-139) | 50  (19-81) | 59  (37-97) | 47  (31-139) |
| **+12**  **months** | 181  (85-308) | 156  (90-245) | 198  (156-308) | 187  (85-267) | **+12**  **months** | 52  (23-102) | 48  (23-92) | 59  (32-99) | 47  (27-102) |
| **LDL**  **[mg/dl]** | **WHOLE COHORT** | **IMA**  **(n=89)** | **NILO**  **(n=59)** | **DASA**  **(n=38)** | **Triglycerides**  **[mg/dl]** | **WHOLE COHORT** | **IMA**  **(n=89)** | **NILO**  **(n=59)** | **DASA**  **(n=38)** |
| **baseline** | 100  (26-205) | 101  (26,2-163,6) | 102  (39,4-155,8) | 80  (26,4-205) | **baseline** | 146  (51-477) | 143  (61-477) | 149  (49-299) | 172  (71-418) |
| **+1**  **month** | 95  (21-190) | 77  (21,2-151,2) | 119  (29-190,4) | 105  (39,2-166,2) | **+1**  **month** | 88  (35-345) | 87  (44-319) | 79  (44-164) | 106  (41-345) |
| **+3**  **months** | 104,2  (20,2-197,6) | 90  (20,4-160,4) | 130*  (37,4-197,6) | 107  (44-197,6) | **+3**  **months** | 105  (34-260) | 104  (37-246) | 99  (34-200) | 141  (45-260) |
| **+6**  **months** | 102  (21-218) | 87  (17-166,6) | 132  (70,6-218) | 101  (34,8-190,6) | **+6**  **months** | 113  (49-280) | 117  (49-280) | 95  (49-243) | 139  (59-272) |
| **+12**  **months** | 106,8  (21-207,2) | 83  (21,4-165) | 120*  (74,4-207,2) | 113  (26-181) | **+12**  **months** | 100  (43-433) | 100  (39-433) | 95  (49-274) | 130  (50-294) |

**Supplemental Table S1.** Median values of total cholesterol, HDL, LDL and tryglicerides. Three-way ANOVA intra and between groups; *p<0.05 nilotinib vs imatinib and dasatinib.

| **SBP**  **[mmHg]** | **WHOLE COHORT** | **IMA**  **(n=89)** | **NILO**  **(n=59)** | **DASA**  **(n=38)** | **DBP**  **[mmHg]** | **WHOLE COHORT** | **IMA**  **(n=89)** | **NILO**  **(n=59)** | **DASA**  **(n=38)** |
| --- | --- | --- | --- | --- | --- | --- | --- | --- | --- |
| **baseline** | 125  (100-170) | 130  (110-170) | 120  (100-140) | 120  (105-150) | **baseline** | 80  (57-90) | 75  (57-90) | 80  (65-80) | 80  (60-90) |
| **+1**  **month** | 125  (105-180) | 120  (115-160) | 130  (100-170) | 128  (100-180) | **+1**  **month** | 75  (50-100) | 80  (67-80) | 76  (50-84) | 70  (69-90) |
| **+3**  **months** | 130  (105-160) | 130  (100-160) | 123  (105-150) | 130  (100-150) | **+3**  **months** | 80  (53-100) | 80  (53-90) | 75  (55-88) | 75  (70-90) |
| **+6**  **months** | 130  (100-170) | 130  (100-170) | 125  (110-160) | 130  (110-170) | **+6**  **months** | 80  (60-90) | 80  (65-90) | 80  (67-85) | 80  (60-90) |
| **+12**  **months** | 130  (100-170) | 130  (110-170) | 130  (100-160) | 133  (110-160) | **+12**  **months** | 80  (50-105) | 79  (60-80) | 80  (60-100) | 80  (60-90) |
| **Glycemia**  **[mg/dl]** | **WHOLE COHORT** | **IMA**  **(n=89)** | **NILO**  **(n=59)** | **DASA**  **(n=38)** | **ABI** | **WHOLE**  **COHORT** | **IMA**  **(n=32)** | **NILO**  **(n=26)** | **DASA**  **(n=16)** |
| **baseline** | 89  (46-252) | 91  (49-150) | 87  (48-140) | 84  (46-252) | **baseline** | 1  (0-1,3) | 1  (1-1,33) | 1  (0-1,3) | 1  (1-1,3) |
| **+1**  **month** | 99  (74-237) | 101  (52-192) | 93  (62-129) | 93  (48-237) | **+1**  **month** | 1,085  (0,9-1,28) | 1  (0,94-1,2) | 1  (0,9-1,28) | 1  (1,08-1,25) |
| **+3**  **months** | 96  (62-236) | 96  (70-165) | 96  (62-138) | 98  (69-236) | **+3**  **months** | 1,08  (0,86-1,2) | 1  (0,86-1,17) | 1  (1-1,2) | 1  (1,04-1,17) |
| **+6**  **months** | 96  (63-183) | 100  (66-149) | 95  (63-174) | 94  (69-183) | **+6**  **months** | 1,1  (0,96-1,28) | 1  (0,96-1,2) | 1  (1,08-1,22) | 1  (1-1,28) |
| **+12**  **months** | 94  (60-206) | 97  (79-175) | 94  (60-206) | 92  (70-150) | **+12**  **months** | 1,04  (0,96-1,16) | 1  (0,96-1,08) | 1  (1-1,16) | 1  (1-1,14) |

**Supplemental Table S2.** Median values of SBP, DBP, glycemia and ABI. Three-way ANOVA; each p is not significant intra and between groups.

| **IL6 [pg/ml]** | **WHOLE**  **COHORT** | **IMATINIB**  **(n=89)** | **NILOTINIB**  **(n=59)** | **DASATINIB**  **(n=38)** |
| --- | --- | --- | --- | --- |
| **baseline** | 2,257  (0-44) | 2,28  (0-44) | 1,66  (0-20,196) | 2,57  (0,2-21,9) |
| **1 month** | 1,339  (0-15,97) | 2,339  (0,25-14,633) | 1,2085  (0-15,97) | 0,83  (0-7,454) |
| **3 months** | 1,1965  (0-82) | 1,392  (0-82) | 1,096  (0-4,57) | 1,0895  (0,17-15,869) |
| **6 months** | 1,15  (0-18,27) | 1,784  (0,06-15,65) | 0,834  (0-18,27) | 1,0905  (0,06-6,91) |
| **12 months** | 1,391  (0-18,08) | 1,804  (0,65-9,86) | 1,2  (0-18,08) | 1,347  (0-14,581) |

**Supplemental Table S3A.** Median IL6 levels in the three TKI cohorts. Three-way ANOVA; each p is not significant intra and between groups.

| **TNFα [pg/ml]** | **WHOLE**  **COHORT** | **IMATINIB**  **(n=89)** | **NILOTINIB**  **(n=59)** | **DASATINIB**  **(n=38)** |
| --- | --- | --- | --- | --- |
| **baseline** | 1,523  (0-18,64) | 1,5775  (0,006-18,641) | 1,396  (0-9,491) | 1,655  (0-17,94) |
| **1 month** | 0,987  (0-8,06) | 1,0395  (0-3,006) | 1,003  (0-8,06) | 0,93  (0-377) |
| **3 months** | 1,12  (0-33,81) | 1,191  (0-33,818) | 1,0345  (0-9,238) | 1,1085  (0-3,9) |
| **6 months** | 0,901  (0-12,83) | 0,9845  (0-6) | 0,84  (0-12,83) | 0,852  (0-3,92) |
| **12 months** | 1,129  (0-30) | 1,157  (0-7,88) | 1,1085  (0-30) | 1,001  (0-18,26) |

**Supplemental Table S3B.** Median TNFα levels in the three TKI cohorts. Three-way ANOVA; each p is not significant intra and between groups.

| **IL10 [pg/ml]** | **WHOLE**  **COHORT** | **IMATINIB**  **(n=89)** | **NILOTINIB**  **(n=59)** | **DASATINIB**  **(n=38)** |
| --- | --- | --- | --- | --- |
| **baseline** | 0,3245  (0-36,77) | 0,4  (0-36,577) | 0,33  (0-26,23) | 0,2  (0-7,536) |
| **1 month** | 0,25  (0-39,21) | 0,687  (0-17,152) | 0,19  (0-39,21) | 0,13  (0-5,943) |
| **3 months** | 0,335  (0-76,89) | 0,62  (0-76,895) | 0,1995  (0-19,06) | 0,06  (0-8,76) |
| **6 months** | 0,25  (0-31,06) | 0,31*  (0-20,772) | 0,165  (0-31,06) | 0,25^#^  (0-9,056) |
| **12 months** | 0,19  (0-17,5) | 0,376*  (0-17,49) | 0,094  (0-14,281) | 0,215^##^  (0-16,62) |

**Supplemental Table S3C.** Median IL10 levels in the three TKI cohorts. Three-way ANOVA intra and between groups; *p=0.008 nilotinib vs imatinib; # p=0.019; ## p=0.01 nilotinib vs dasatinib.

| **oxLDL [pg/ml]** | **WHOLE**  **COHORT** | **IMATINIB**  **(n=89)** | **NILOTINIB**  **(n=59)** | **DASATINIB**  **(n=38)** |
| --- | --- | --- | --- | --- |
| **baseline** | 136,19  (14,07-3595,85) | 109,48  (14,07-3115,3) | 153,72  (14,82-3595,85) | 101,04  (27,83-2168,9) |
| **1 month** | 99,42  (16,51-2546,64) | 93,01  (16,51-2452,09) | 123,82  (37,86-2546,64) | 88,42  (17,46-1854,29) |
| **3 months** | 103,92  (1,27-6911,9) | 90,6  (2,72-2486,9) | 154,67  (46,84-6911,9) | 100,995  (1,27-1039,16) |
| **6 months** | 93,52  (9,97-2560,9) | 84,3  (9,97-2560,9) | 164,8  (37,76-1003,6) | 70,37  (21,41-517,01) |
| **12 months** | 90,83  (5,74-2392,46) | 82,005  (5,74-1884,65) | 171,68*  (42,41-2392,46) | 70,29  (30,22-993,02) |

**Supplemental Table S4.** Median oxLDL levels in the three TKI cohorts. Three-way ANOVA intra and between groups; *p=0.026 nilotinib vs imatinib and dasatinib.
